# Dapagliflozin Improved Cardiac Function and Structure in Diabetic Patients with Preserved Ejection Fraction: Results of a Single Centre, Observational Prospective Study

**DOI:** 10.3390/jcm12206698

**Published:** 2023-10-23

**Authors:** Marcelino Cortés, Oscar Lorenzo, Jairo Lumpuy-Castillo, Sacramento Martínez-Albaladejo, Mikel Taibo-Urquía, Ana María Pello, Antonio José Bollas, Miguel Orejas, Miguel Ángel Navas, Ester Macia, María Esther Martínez, Andrea Rueda, Jose Tuñón

**Affiliations:** 1 Cardiology Department, Fundación Jiménez Díaz Hospital, 28040 Madrid, Spain; mikel.taibo@quironsalud.es (M.T.-U.); ampello@quironsalud.es (A.M.P.); antonio.bollas@quironsalud.es (A.J.B.); morejaso@fjd.es (M.O.); manavasl@quironsalud.es (M.Á.N.); emacia@quironsalud.es (E.M.); memartinezf@quironsalud.es (M.E.M.); andrea.ruedal@quironsalud.es (A.R.); jtunon@quironsalud.es (J.T.); 2 Laboratory of Diabetes and Vascular Pathology, IIS-Fundación Jiménez Díaz, Universidad Autónoma, 28040 Madrid, Spain; olorenzo@fjd.es (O.L.); jlumpuy@gmail.com (J.L.-C.); sacramento.martinez@quironsalud.es (S.M.-A.); 3 Biomedical Research Network on Diabetes and Associated Metabolic Disorders (CIBERDEM), Carlos III National Health Institute, 28029 Madrid, Spain

**Keywords:** sodium-glucose cotransporter type 2 inhibitors, dapagliflozin, speckle tracking, biomarkers, echocardiography

## Abstract

Sodium-glucose cotransporter inhibitors (SGLT2i) have demonstrated a reduction in cardiovascular events in diabetes and heart failure (HF). The mechanisms underlying this benefit are not well known and data are contradictory. The purpose of this study is to analyse the effect of dapagliflozin on cardiac structure and function in patients with normal ejection fraction. Between October 2020 and October 2021, we consecutively included 31 diabetic patients without prior history of SGLT2i use. In all of them, dapagliflozin treatment was started. At inclusion and during six months of follow-up, different clinical, ECG, analytical, and echocardiographic (standard, 3D, and speckle tracking) variables were recorded. After a follow-up period of 6.6 months, an average reduction of 18 g (*p* = 0.028) in 3D-estimated left ventricle mass was observed. An increase in absolute left ventricle global longitudinal strain (LV-GLS) of 0.3 (*p* = 0.036) was observed, as well as an increase in isovolumetric relaxation time (IVRT) of 10.5 ms (*p* = 0.05). Moreover, dapagliflozin decreased the levels of plasma creatin-kinase (CK-MB) and atrial natriuretic peptide (ANP). In conclusion, our data show that the use of SGLT2i is associated with both structural (myocardial mass) and functional (IVRT, LV-GLS) cardiac improvements in a population of diabetic patients with normal ejection fraction.

## 1. Introduction

Heart failure (HF) continues to be a prevalent and relevant clinical problem. The current prevalence of HF in the adult population is estimated to be 1–2% [1]. Despite great advances in recent years in treatment and management of these patients, morbidity and mortality associated with HF remain high [2]. Diabetes mellitus (DM) represents a risk factor for HF, with a higher rate of events and mortality in this group of patients [3], and can also lead to the entity known as diabetic cardiomyopathy.

Sodium-glucose cotransporter type 2 (SGLT2) inhibitors (SGLT2i) are a new family of drugs developed for treatment of type 2 DM [4]. Initial studies in diabetic patients with some of these drugs showed a striking clinical benefit in cardiovascular diseases, particularly in regard to HF [5,6,7]. Following these results, several relevant trials have analysed the clinical effect of SGLT2i directly in populations of HF patients with reduced and preserved ejection fraction, with and without DM [8,9,10,11,12], resulting in the inclusion of both empagliflozin and dapagliflozin as first-line treatment for HF [13].

The mechanisms underlying this clinical benefit are not well known. The benefit cannot be explained solely by the hypoglycaemic effect, reduction in atherosclerotic events, beneficial effect on renal function, or the reduction in body weight and blood pressure associated with the use of SGLT2i [14]. Different studies suggest that the reduction of HF hospitalisation could be explained by a structural and functional improvement of the left ventricle, but their results give rise to differing conclusions. [15,16,17,18,19,20,21,22]. The use of speckle tracking imaging techniques is especially useful for detecting subtle and early changes in cardiac function, which could provide useful information. However, little or no data have been published to date using these imaging techniques to analyse the effect of SGLT2i on the heart. Additionally, antioxidant, anti-inflammatory, anti-fibrotic, and anti-hypertrophic actions as well as bioenergetic changes have been associated with SGLT2i administration [23]. In this sense, the use of circulating biomarkers that are representative of cardiac cellular events could help to better understand the mechanisms involved.

In short, although the efficacy and safety of SGLT2i have been demonstrated in different clinical trials, the mechanisms underlying their clinical benefits are not yet fully understood, resulting in an incomplete understanding of the effect of these drugs. In our work, we analysed the effect of dapagliflozin on a population of diabetic patients with preserved ejection fraction by means of a deep structural and functional study with imaging techniques (including speckle tracking imaging) and specific biochemical markers.

## 2. Material & Methods

### 2.1. Patients and Study Design

We carried out a single-centre, observational prospective study. From October 2020 to October 2021, we prospectively enlisted 31 patients from cardiology or endocrinology outpatient clinics at our centre for whom dapagliflozin treatment was considered on the basis of clinical criteria. The inclusion criteria were the following: (1) type 2 DM diagnosed according to the 2019 recommendations of the American Diabetes Association [24]; (2) glycosylated haemoglobin >6.5%; (3) LV ejection fraction (LVEF) greater than or equal to 50% as measured by echocardiography; (4) sinus rhythm; (5) one or more of the following echocardiogram findings: any degree of diastolic dysfunction, LV hypertrophy (defined as indexed LV mass ≥115 g/m^2^ in men, and ≥95 g/m^2^ in women), and/or left atrial dilatation (defined as indexed volume >34 mL/m^2^); and (6) age >18 years.

The major exclusion criteria were: (1) previous treatment with SGLT2i; (2) previous intolerance or allergy to SGLT2i; (3) glomerular filtration rate (GFR) lower than 60 mL/min/1.73 m^2^; (4) acute coronary syndrome within the last three months; (5) relevant valve heart disease, hypertrophic cardiomyopathy, diagnosed cardiac amyloidosis, complex congenital heart disease, or other specific heart diseases; and (6) any other medical condition considered inappropriate by the study physician.

Our study included two visits. At the baseline visit (pre-treatment, first visit), all patients underwent a clinical assessment, anthropometric measurements (height and weight) and an echocardiographic study. Data on comorbidities, pharmacological treatment, and diagnostic techniques (electrocardiography and echocardiography) were collected. We also obtained and preserved blood and urine samples. After the first visit, all patients received 10 mg of dapagliflozin daily for a minimum period of six months, according to habitual clinical practice [3]. After six to nine months, a second visit was performed, in which blood and urine collection and an echocardiogram were repeated.

The study design and protocol were revised and approved by the Clinical Research Ethics Committee of our institution (Ref. PIC192-19_FJD), and informed written consent was obtained from all participants. This investigation was conducted in accordance with the principles outlined in the Declaration of Helsinki.

### 2.2. Echocardiography

Comprehensive transthoracic echocardiography was performed using a commercially available system (EPIQ CVx 7.0.3, Philips Healthcare, Best, The Netherlands) equipped with a ×5–1 xMATRIX array transducer, according to a standardised protocol. Chamber size and quantifications were measured according to guidelines of the American Society of Echocardiography (ASE) and the European Association of Cardiovascular Imaging (EACVI) [25,26]. Moreover, a specific 3D echocardiogram study of LV and left atrium (LA) was performed. We obtained standard measurements such as LV end-diastolic diameter; LA diameters; interventricular septum and posterior wall thickness; E, A, e’ and a’ waves; isovolumetric relaxation time (IVRT); TAPSE;S’ wave; and VD dimensions, among others. LVEF, indexed LV mass, and indexed LA volumes were measured using 3D-echocardiography. Strain imaging was performed with speckle tracking software, which measured LV, LA, and right ventricle (RV) strain in accordance with specific consensus documents and position papers published by the EACVI (European Association of Cardiovascular Imaging) and the ASE (American Society of Echocardiography) [27,28]. The longitudinal strain of the 17 segments of the LV was measured using two-, three-, and four-chamber apical views. LV peak longitudinal strain and global longitudinal strain (LV-GLS) values for each patient were calculated automatically by the software. Tracking points were placed automatically by the software on an end-systolic and end-diastolic frame in each view, with slight manual changes made if deemed necessary. RV longitudinal strain and LA strain analysis were performed. Strain analysis of the LA was performed in the four-chamber view, using the QRS complex as the baseline reference point. We also measured the following parameters: peak atrial longitudinal strain (PALS), peak atrial contraction strain (PACS), and LA strain during the conduit phase (LACS). Strain analysis of the RV was performed in the RV-focused apical four-chamber view. The RV endocardial border was traced at both the end-diastolic and end-systolic frames automatically by the software, with slight manual changes made as needed. We measured the following parameters: right ventricular four-chamber strain (RV4CSL) and right ventricular free-wall longitudinal strain (RVFWSL). RV4CSL included both the RV free wall and interventricular septum segments. RV free wall strain (RVFWSL) was defined as the strain value at the RV free wall.

The analysis of all cases was performed by only one observer, an expert cardiologist (M.C.) experienced in strain measuring and 3D echocardiography, using specific speckle tracking software (AutoStrain and Dynamic HeartModel, Epiq CVx version 7.0.5, Philips Healthcare, Best, The Netherlands).

### 2.3. Biochemical Analysis

Serum, plasma, and urine samples were extracted and stored (at −80 °C) at two points during the study. First, at the inclusion of the patient in the study, before starting treatment with dapagliflozin, and again after six to nine months of follow-up. We measured the usual parameters in urine and blood (complete blood count, kidney function, liver function, lipid profile, urine sediment, proteins, etc.). Additionally, we analysed the levels of the following specific biomarkers related to possible cardiac remodelling responses: monocyte chemoattractant protein-1 (MCP-1) and interleukin-6 (IL-6) as heart pro-inflammatory biomarkers [29], cardiotrophin-1 as a marker of myocardial hypertrophy [30], and heart-fatty acid binding protein (H-FABP) as a marker of myocardial steatosis [31]. We also measured known biomarkers such as N-terminal prohormone of brain natriuretic peptide (NT-proBNP) as markers for HF, myocardial hypertrophy, and vasodilation; high-sensitivity C-reactive protein (hsCRP), matrix metallopeptidase-2 and -9 (MMT-2 and MMP-9), and T-cell immunoglobulin and mucin domain-1 (TIM-1) as markers of inflammation; atrial natriuretic peptide (ANP) as a marker of myocardial hypertrophy and vasodilation; galectine-3 (GAL-3) as a marker of inflammation and fibrosis; and creatine kinase MB (CK-MB) as a marker of myocardial injury. The enzyme-linked immunosorbent assays (ELISA) were performed to quantify ANP (DANP00, Bio-Techne, Minneapolis, MN, USA), MMP-2 (MMP200, Bio-Techne, Minneapolis, MN, USA) and FABP3 (DY1678, Bio-Techne, Minneapolis, MN, USA). The technique was carried out following the manufacturer’s instructions. The absorbance reading was taken at 450 nm with a 570 nm wavelength correction in the plate reader (EnSpire^®^ Multimode Reader Perkin Elmer, Waltham, MA, USA). MCP-1 (SPCKA-PS-008756), IL-6 (SPCKA-PS-008755), TIM-1 (SPCKA-PS-008754), HS-CRP (SPCKB-PS-000200), MMP-9 (SPCKB-PS-000661), and Galectin-3 (SPCKB-PS-000490) were quantified by the ELLA™ automated immunoassay system (Bio-Techne, Minneapolis, MN, USA). CK-MB was measured by the Analytical Laboratory of Fundación Jiménez Díaz University Hospital (ref.: 1896836, VTROS Immunodiagnostic Products, NJ, USA).

### 2.4. Follow-Up, Adverse Events and Outcomes

Clinical outcomes and adverse events were monitored afterwards and during the follow-up. The outcomes analysed in our study were admission due to HF and death from any cause. HF admission was defined as admission to a healthcare facility lasting more than 24 h due to the onset or worsening of HF symptoms and followed by specific treatment for HF. Adverse events recorded were hypoglycaemia, urinary or genital infections, and pharmacological changes, among others. All these clinical events during follow-up were collected from patient visits or electronic health records.

### 2.5. Statistical Analysis

Data were subjected to descriptive statistical analyses with frequency measurements (absolute frequencies and percentages) for qualitative variables and with mean and standard deviation or median and interquartile range for quantitative variables. We performed a comparative analysis of the different variables at baseline and after six to nine months of treatment with dapagliflozin. The analysis of the different variables was performed using the paired Student’s *t*-test when the distribution of the variable was assumed to be normal and the Mann–Whitney U-test (Wilcoxon) when the distribution of the variable was not assumed to be normal. A *p*-value of less than 0.05 was considered statistically significant. We randomly selected eight patients from the study cohort and analysed the intraobserver reproducibility of the IVRT, E, and e’ wave measurements, among others. A variability analysis of variables such as myocardial mass, LV strain, or LA volume was not performed because these variables were obtained automatically by the echocardiography software. The intraclass correlation coefficients (absolute agreement) of IVRT, E, and e’ waves were 0.9025, 0.961, and 0.877, respectively. To ensure reproducibility, these variables were measured again in the same sample of eight studies by a second experienced operator (M.T.) in a blinded fashion to determine interobserver variability. The interobserver intraclass correlation coefficients of IVRT, E, and e’ were 0.802, 0.993, and 0.965, respectively.

The sample size was estimated for this study. By considering an alpha risk of 0.05 (two-sided test), beta risk of 0.2, standard deviation of 3.5, minimum expected difference of 2.2, and a dropout rate of 0.1, the GRANMO software (v7.2, Barcelona, Spain) suggested a minimum of 23 patients. The calculation of the standard deviation and the minimum expected difference on echocardiographic variables was based on data from previous reports [32,33].

Statistical analyses were performed with SPSS version 22.0 (SPSS, Inc., Chicago, IL, USA).

## 3. Results

### 3.1. Characteristics and Follow-Up of the Study Population

**Figure 1:**
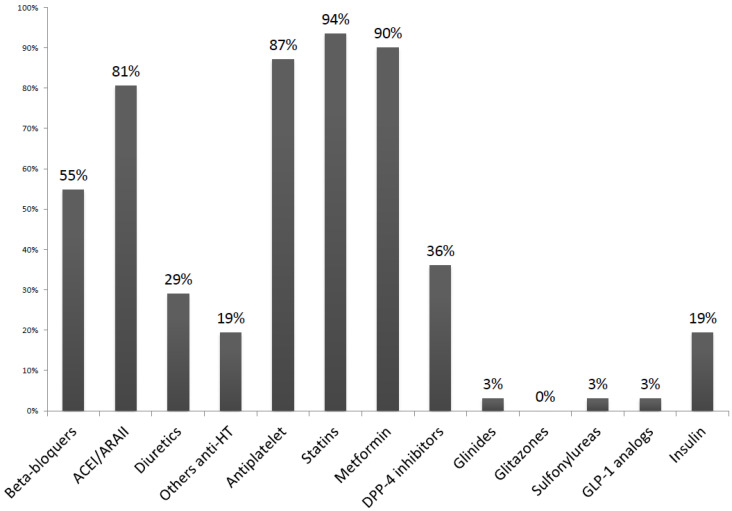
Pharmacological treatment of the study population at inclusion. ACEI: angiotensin-converting enzyme inhibitors; ARB: angiotensin receptor blocker; HT: hypertension.

A total of 31 type 2 DM patients were enrolled in the study. The average age of our population was 66.4 years (±8.4), and 90% of patients were male. The average time elapsed since diagnosis of diabetes was 10.6 years (±7.8). Although the presence of HF was not described as an exclusion criterion, none of the patients included in the study had a previous history of HF. The baseline characteristics of our study population and the baseline pharmacological treatment are shown in Table 1 and Figure 1, respectively.

After an average follow-up period of 6.6 (±0.8) months, dapagliflozin had to be withdrawn in five patients due to repeated genital or urinary infections (three patients) or at the patient’s behest. Two patients refused to undergo the echocardiogram or laboratory tests at the end of follow-up, although they were in the group of five for whom dapagliflozin was discontinued. The remaining 29 patients completed the study.

Therefore, we performed a comparative analysis before and after treatment with dapagliflozin in those patients who continued taking the drug throughout the follow-up (26 patients). This analysis showeda significant body weight reduction (*p* < 0.001) after 6.6 months of treatment, as expected. We also observed a decrease in plasma glucose (*p* = 0.02) and a tendency of reduction of glycosylated haemoglobin, with no significant reduction in glomerular filtration. Treatment with dapagliflozin significantly reduced proteinuria (*p* = 0.005) and blood ferritin levels (*p* = 0.018), and increased glycosuria (not shown), HDL cholesterol (*p* = 0.031), and haemoglobin (*p* = 0.023). Table 2 shows the comparative analysis of the different variables included in our study.

### 3.2. Echocardiographic Data: Functional and Structural Changes

**Figure 2:**
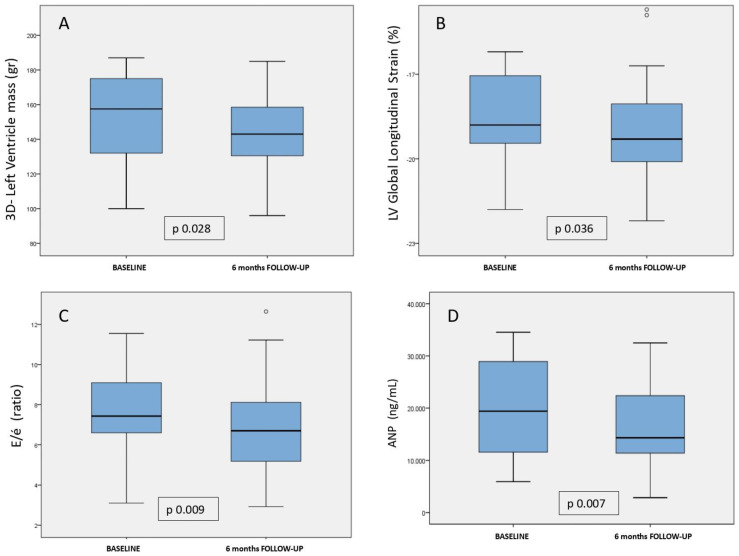
Changes in left ventricle mass, global longitudinal strain, E/e’ ratio, and ANP after dapagliflozin treatment. Dapagliflozin is associated with greater reduction in 3D-left ventricular mass (**A**), left ventricular global longitudinal strain (**B**), with the E/e’ ratio (**C**), and with the ANP plasma levels (**D**). Graphs represent mean and 95% confidence interval.

We performed a comparative analysis of echocardiographic variables before and after treatment with dapagliflozin in patients who continued taking the drug throughout follow-up. We observed an average reduction in 3D-estimated LV mass of 18 g (*p* = 0.028) after six months of treatment. Also, an increase in absolute LV-GLS of 0.3 (*p* = 0.036) was observed, as well as an increase in IVRT of 10.5 ms (*p* = 0.05), an increase in TAPSE of 2.1 mm (*p* = 0.01), and a significant decrease in the E/e’ ratio of 0.8 (*p* = 0.009). A mild increase in e’, LA, and VD strain and a decrease in LA indexed volume were noted, although differences did not achieve statistical significance. Table 2 and Figure 2 show the echocardiographic variables analysed in our study before and after dapagliflozin.

### 3.3. Biochemical Analysis: Changes in Hypertrophy Biomarkers

Following the described methodology, we analysed plasma and urine samples from our study population at baseline and after follow-up in patients who continued to take dapagliflozin during follow-up. We observed a significant reduction in ANP (biomarker with vasodilatation and anti-hypertrophic functions, *p* = 0.007) and CK-MB levels (biomarker associated with myocardial injury, *p* = 0.006) after dapagliflozin treatment. The comparative analysis showed a trend towards an increase of pro-inflammatory biomarkers (MCP-1, hsCRP, GAL-3, MMP-2/9) without reaching statistical significance. Other biomarkers such as H-FABP also showed slight, non-statistically significant decreases at the end of follow-up. Table 2 and Figure 2 show the comparative analysis of the biochemical variables included in our study.

### 3.4. Association between Biomarkers and Echocardiographic Variables

**Figure 3:**
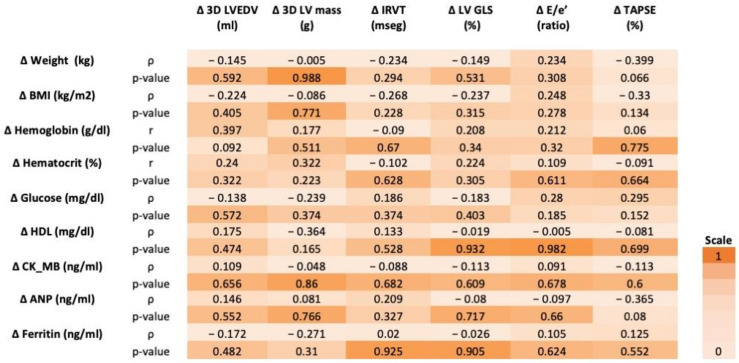
Association between biomarkers and echocardiographic variables: data matrix.∆: absolute difference from baseline; ρ: Spearman’s rho (correlation coefficient); r = Pearson’s r (correlation coefficient). ANP: atrial natriuretic peptide; BMI: body mass index; CK-MB: creatinine phosphokinase-MB; IVRT: isovolumic relaxation time; LV: left ventricle; LVEDV: LV end-diastolic volume; LV GLS: LV global longitudinal strain.

We performed a specific analysis to evaluate the possible relationships that could be established between those variables in which a statistically significant change was observed after the period of dapagliflozin treatment. Specifically, we studied the possible association between all those clinical and analytical variables with statistically significant changes with respect to the echocardiographic variables that in turn showed significant differences. The analysis did not show any association between the different clinical and analytical variables assessed and the echocardiographic variables. Thus, the reduction in weight or glycemia and the reduction in ANP and circulating CK-MB after 6.6 months of dapagliflozin treatment failed to explain the improvement in diastolic function or cardiac hypertrophy in our population. Figure 3 shows the data matrix with the results of this analysis.

## 4. Discussion

Our study shows that the use of dapagliflozin in diabetic patients with preserved ejection fraction and without HF but with data suggesting incipient functional and/or structural damage is associated with an improvement in functional heart parameters (both systolic and diastolic function), as well as a reduction in myocardial mass, in just 6.6 months of treatment. Our data could help explain the clinical benefits of SGLT2i in HF.

HF continues to be a prevalent and relevant issue [1,34], with high mortality and morbidity [2]. This problem is accentuated in diabetic patients, being one of the main causes of hospital admission in this population [35,36]. Moreover, DM is a poor prognosis factor in HF, as it further elevates the rate of events and mortality in the population [3]. In this regard, it has been shown in test animals and patients that type 2 DM produces inflammatory phenomena, cardiac hypertrophy, and steatosis, which can result in cellular apoptosis and necrosis and cardiac remodelling, profoundly affecting cardiac function [35] and potentially leading to direct myocardial damage in relation to DM, producing the condition known as diabetic cardiomyopathy (DCM) [37]. DCM, together with other factors such as arterial hypertension and coronary artery disease, causes a significant percentage of diabetic patients to present both systolic and diastolic functional alterations, even with preserved ejection fraction [38,39].

SGLT2i acts in the proximal tubule of the nephron, increasing urinary glucose excretion [40]. However, safety studies of these drugs in diabetic patients showed a striking beneficial clinical effect on cardiovascular disease, particularly on HF: empagliflozin showed a significant reduction in mortality (5.7% vs. 8.3%) and HF hospitalisations (2.7% vs. 4.1%) [5]; canagliflozin significantly reduced the combined end point of cardiovascular mortality, stroke, or myocardial infarction, as well as a marked reduction in HF hospitalisation (HR 0.68; IC 95%, 0.51–0.90) [6]; and dapagliflozin showed a significant reduction in HF hospitalisations (HR 0.73; IC 95% 0.61–0.88) [7]. Following these studies, the effect of SGLT2i was studied also directly in populations of patients with HF with reduced ejection fraction, both with and without DM. DAPA-HF showed a significant reduction in both HF hospitalisations (HR 0.7; IC 95%, 0.59–0.83) and all-cause mortality (HR 0.83; IC 95%, 0.71–0.97) with dapagliflozin [8]; and EMPEROR-Reduced showed a significant reduction (with empagliflozin) of a combined end-point of cardiovascular death and HF hospitalisation [9]. More recently, several studies have been published showing clinical benefit of SGLT2i in HF with preserved ejection fraction [10,11,12], as well as several meta-analyses that confirm the clinical benefit of SGLT2i in HF [41,42]. All this evidence has led to SGLT2i being included in clinical practice guidelines as one of the four pillars of HF with reduced ejection fraction [13].

The mechanisms that explain the aforementioned clinical benefits are not well known. The alterations in myocardial function and structure that are commonly found in patients with DM [37,43], together with the relative scarcity of reduction in acute arteriosclerotic events that SGLT2i use causes, has suggested that the reduction in HF hospitalisations might be caused by an improvement in LV function and not a reduction in atherosclerotic burden [15]. This hypothetical improvement in LV structure and function might occur in response to indirect mechanisms on the myocardium (haemodynamic, metabolic, etc.) rather than to a direct mechanism, considering the absence of SGLT2 receptors in the heart [44]. In this sense, different authors have studied the changes in LV structure and function derived from the use of SGLT2, using imaging techniques such as echocardiography and magnetic resonance imaging (MRI). These studies included few patients but were able to show that just a few months (3–6) of treatment produced an increase in diastolic function and a reduction of LV mass, without a clear improvement in ejection fraction or LV volumes [16,17,18,19]. However, Cohen et al. showed a reduction in LV volume as measured with MRI after six months of treatment with empagliflozin [20]. There is a dearth of data with respect to myocardial strain in these patients. Speckle tracking echocardiography has shown its usefulness in being able to detect initial alterations of cardiac function, which could advance diagnosis and treatment of patients [45,46,47,48,49,50]. In particular, speckle tracking echocardiography can identify early functional improvement or deterioration, allowing the detection of cardiac alterations in type 2 DM patients and the understanding of SGLT2i in the prevention of structural deterioration of the LV.

In our study, we performed an in-depth imaging study with speckle tracking echocardiography in a population of diabetic patients without prior SGLT2i treatment, completing the obtained results with a wide array of cardiac biomarkers of fibrosis, hypertrophy, and necrosis. Dapagliflozin was associated with early improvement (at 6.6 months) in both cardiac structure and function in a population of diabetic patients with normal ejection fraction. It also induced a significant reduction in indexed LV mass, as well as a significant improvement in LV systolic function as evidenced by an increase in the LV-GLS absolute value, and an improvement in LV diastolic function as reflected by a significant increase in IVRT and decrease in the E/e’ ratio. This last finding is particularly relevant, considering that diastolic function deterioration is the main cardiac alteration associated with DM [37].

In addition, a significant reduction in ANP levels was observed in our study population. ANP regulates salt-water balance and blood pressure by promoting renal sodium and water excretion and stimulating vasodilation. Furthermore, ANP has an anti-hypertrophic effect on the heart, independent of its systemic anti-hypertensive effect [51]. This effect could be related to a reduction in biological hypertrophic responses in the heart, in line with our result of a diminution in myocardial mass, as well as lessening in LA pressure after improving systolic and diastolic function. We also observed a significant decrease in CK-MB levels in our study population, which indicates a reduction in myocardial injury. As with ANP, the improvement in diastolic function leads to less myocardial overload, which could explain the reduction in myocardial injury. These effects must only be considered possible etiological hypotheses of the findings, and specific studies are needed to determine the exact relationship between dapagliflozin and the production of ANP and release of CK-MB.

We didnot find significant changes in the rest of the associated analysed biomarkers, especially in regard to fibrosis or inflammation markers, but we have observed a significant increase in haemoglobin and haematocrit. This is in line with previous observations that SGLT2i use leads to an increase in erythropoietin levels and haemoconcentration (related to the diuretic effect) [23,52]. A higher haemoglobin level could enhance oxygen delivery to the myocardium, theoretically improving its function, but the low increase in haemoglobin does not appear to be sufficient to explain fully the improvement in function. In addition, at the urinary level, we observed the presence of marked glycosuria (inherent to the renal effect of SGLT2i), as well as a significant reduction in patients with proteinuria, which we related to the nephroprotective effect associated with SGLT2i [14].

All the results point to an improvement in intracardiac pressures as a basic mechanism in the clinical improvement associated with dapagliflozin, in favour of all other mechanisms. These effects could be explained by the haemodynamic effect of dapagliflozin derived from its renal effect (increasing diuresis and favouring a reduction in blood pressure, resulting in a reduction in cardiac preload and afterload). Nonetheless, the improvement in contractile function, as evidenced by a significant increase in LV-GLS, also suggests a more direct effect on the cardiomyocyte. This supports theories that postulate a direct effect of SGLT2i on cellular metabolism of these cells [53], improvement of their contractility, and reduction of myocardial damage, a possibility that is further supported by our finding of a significant reduction in CK-MB in our test population.

Nevertheless, this study has three limitations. Firstly, although it was prospective, it was an uncontrolled observational study with a small number of patients included (with a predominance of males), and larger randomised controlled trials are, therefore, needed. Secondly, we could not verify whether the improvement in diastolic function was solely attributable to dapagliflozin or to any particular effect of dapagliflozin (diuretic effect, weight loss, etc.) or other associated pharmacological treatment. Lastly, the relationship between the administration of dapagliflozin and cardiovascular events could not be verified due to the short research period.

## 5. Conclusions

The use of dapagliflozin for at least six months is associated with cardiac improvement, both structural (myocardial mass) and functional (IVRT, LV-GLS) in a population of diabetic patients even with normal ejection fraction, reducing CK-MB and ANP levels, which could have relevance in improving the clinical outcome of our patients. Furthermore, although our results do not show the initial cause of this structural and functional improvement, they do provide relevant data for other authors working on the different aetiological hypotheses currently under development.

## Figures and Tables

**Table 1:** Demographic and clinical characteristics of study population.

Variables	n = 31
Age (mean (±SD))	66.4 (±8.4)
Male (n (%))	28 (90.3)
Hypertension (n (%))	22 (71.0)
Dyslipidaemia (n (%))	24(77.4)
Tobacco (n (%))	27 (87.1)
Obesity (n (%))	8 (25.8)
Cerebrovascular disease (n (%))	1 (3.2)
Peripheral vascular disease (n (%))	3 (9.7)
Chronic pulmonary disease (n (%))	3 (9.7)
Glomerular filtration (mL/min/1.73 m^2^ (±SD))	83.2 (±13.2)
Ischemic heart disease (n (%))	24 (77.4)
QRS >120 (n (%))	5 (16.1)

**Table 2:** Comparative analysis before and after 6 months of follow-up.

	Baseline (±SD or IR)	Dapagliflozin (±SD or IR)	*p* Value
Biochemichal variables (*)			
Hemoglobin (g/dL)	14.4 ± 1.4	14.9± 1.8	0.023
Hematocrit (%)	42.9 ± 3.8	45.1 ± 4.5	<0.001
Creatinin (mg/dL)	0.9 (0.2)	1 (0.4)	NS
GF (mL/min/1.73 m^2^)	86.2 (22.2)	84.4 (26.0)	NS
Potassium (mmol/L)	4.5 ± 0.4	4.5 ± 0.4	NS
Cholesterol (mg/dL)	136.5 (58)	135 (37)	NS
LDL cholesterol (mg/dL)	61 (31)	64.5 (18)	NS
HDL cholesterol (mg/dL)	47.5 (14)	50 (19)	0.031
Glucose (mg/dL)	129.5 (46.5)	124 (32.25)	0.02
Glyc. hemoglobin (%)	6.9 (0.8)	6.8 (0.9)	NS
Ferritin (ng/mL)	55.5 (183)	41.5 (123)	0.018
Proteinuria (%)	17.4%	8.7%	0.005
Urine pH	5.7 ± 0.7	5.6 ± 0.7	NS
NT-ProBNP (pg/mL)	52.4 (76.5)	51.5 (116)	NS
CK-MB (ng/mL)	1.34 (1.1)	1.32 (1.5)	0.006
MCP-1 (pg/mL)	212 (95)	228 (88)	NS
IL-6 (pg/mL)	3.26 (2.78)	2.68 (2.47)	NS
MMP-9 (ng/mL)	489.9 (470.5)	554.9 (473.7)	NS
hs-CRP (mg/mL)	1.05 (2.07)	1.26 (1.86)	NS
GAL-3 (ng/mL)	6.9 ± 1.7	7.5 ± 2.4	NS
TIM-1(pg/mL)	91.3 (67.7)	103 (72.5)	NS
ANP (ng/mL)	19.4 (17.99)	14.3 (12.29)	0.007
MMP-2 (ng/mL)	277.1 ± 100.2	297.2 ± 88.7	NS
H-FABP (ng/mL)	1.01 (0.60)	0.85 (0.8)	NS
Clinical variables (*)			
Body weight (kg)	80 (20)	74.0 (22.0)	<0.001
BMI (kg/m^2^)	27.68 (5.02)	26.5 (5.2)	<0.001
Ecocardiographic variables (*)			
LVEF (%)	59.8 ± 3.5	60.5 ± 3.0	NS
3D LVEDV (mL)	116.5 (65.9)	118 (51)	NS
3D LVESV (mL)	48 (25.8)	48 (19)	NS
3D LV mass (g)	162 (48)	144 (35)	0.028
LV-GLS (%)	−19 (2.9)	−19.3 (2.3)	0.036
LA 3D-volume (mL/m^2^)	37.7 ± 10.1	37.8 ± 13.4	NS
PALS(%)	34.1 ± 12.8	32.5 ± 9.7	NS
PACS(%)	−16.7 ± 8.2	−16.1± 7.2	NS
LACS(%)	−17.0 (11.3)	−13.9 (8.1)	NS
TAPSE (mm)	20.9 ± 3.3	23 ± 3.4	0.01
RVFWSL(%)	−23.1 ± 5.8	−23.4 ± 3.5	NS
RV4CSL (%)	−19.1 ± 4.1	−18.4 ± 2.4	NS
é wave (cm/s)	8.3 ± 1.9	9.1 ± 2.3	NS
E/é (ratio)	7.4 (2.5)	6.6 (3.2)	0.009
IRVT (ms)	89.5 ± 18.9	100.0 ± 17.6	0.05

* Values of variables (baseline and follow-up) are expressed as mean ± SD, or median (IR), depending on whether their distribution is normal or not. ANP: atrial natriuretic peptide; BMI: body mass index; CK-MB: creatinine phosphokinase-MB; GAL-3: Galectin-3; GF: glomerular filtration; H-FABP: heart-fatty acid binding protein; hs-CRP: high sensitivity C-reactive protein; IL-6: interleukin-6; IR: interquartile range; IVRT: isovolumic relaxation time; LA: left atrium; LV: left ventricle; LVEF: LV ejection fraction; LVEDV: LV end-diastolic volume; LVESV: LV end-systolic volume; LV-GLS: LV global longitudinal strain; MCP-1: monocyte chemoattractant protein-1; MMP-2: matrix metallopeptidase 2; MMP-9: matrix metallopeptidase 9; PALS: peak atrial longitudinal strain; PACS: peak atrial contraction strain; LACS: LA strain during the conduit phase; RVFWSL: right ventricular free-wall longitudinal strain; RV4CSL: right ventricular four-chamber strain; SD: standard deviation; TIM-1: T-cell immunoglobulin and mucin domain 1. NS: non-significant

## Data Availability

The datasets used and analysed during the current study are available from the corresponding author on reasonable request.
